# A computational comparison of radiofrequency and pulsed field ablation in terms of lesion morphology in the cardiac chamber

**DOI:** 10.1038/s41598-022-20212-9

**Published:** 2022-09-27

**Authors:** Mario Gómez-Barea, Tomás García-Sánchez, Antoni Ivorra

**Affiliations:** 1 Department of Information and Communication Technologies, Universitat Pompeu Fabra, 08018 Barcelona, Spain; 2 Serra Húnter Fellow Programme, Universitat Pompeu Fabra, 08018 Barcelona, Spain

**Keywords:** Cardiology, Biomedical engineering, Computational science

## Abstract

Pulsed Field Ablation (PFA) has been developed over the last years as a novel electrical ablation technique for treating cardiac arrhythmias. It is based on irreversible electroporation which is a non-thermal phenomenon innocuous to the extracellular matrix and, because of that, PFA is considered to be safer than the reference technique, Radiofrequency Ablation (RFA). However, possible differences in lesion morphology between both techniques have been poorly studied. Simulations including electric, thermal and fluid physics were performed in a simplified model of the cardiac chamber which, in essence, consisted of a slab of myocardium with blood in motion on the top. Monopolar and bipolar catheter configurations were studied. Different blood velocities and catheter orientations were assayed. RFA was simulated assuming a conventional temperature-controlled approach. The PFA treatment was assumed to consist in a sequence of 20 biphasic bursts (100 µs duration). Simulations indicate that, for equivalent lesion depths, PFA lesions are wider, larger and more symmetrical than RFA lesions for both catheter configurations. RFA lesions display a great dependence on blood velocity while PFA lesions dependence is negligible on it. For the monopolar configuration, catheter angle with respect to the cardiac surface impacted both ablation techniques but in opposite sense. The orientation of the catheter with respect to blood flow direction only affected RFA lesions. In this study, substantial morphological differences between RFA and PFA lesions were predicted numerically. Negligible dependence of PFA on blood flow velocity and direction is a potential important advantage of this technique over RFA.

## Introduction

Nowadays, radiofrequency ablation (RFA), together with cryoablation, are the most extended non-pharmacological techniques used in the ablation of myocardial tissue for the treatment of cardiac arrhythmias. RFA is extensively used in patients with the most prevalent arrhythmia: atrial fibrillation (AF)^1,2^. In particular, pulmonary vein isolation (PVI) using RFA has been shown to be an effective treatment option in patients with AF, obviating the need for antiarrhythmic and anticoagulation drugs. Cardiac ablation therapies reduce both the recurrence of AF events and of the hospitalization time and improve the quality of life in patients^1^.

Radiofrequency ablation is based on producing thermal damage to a region of tissue as consequence of focal Joule heating by the passage of alternating electric currents. Such heating is typically induced by delivering, through electrodes mounted on catheters, sinusoidal electric currents with a frequency of around 500 kHz^3,4^. To control heating, RFA systems use three different strategies: constant voltage, constant power or constant temperature^5^. The first strategy consists in maintaining a constant voltage value across the electrodes in contact with the tissue. The second one consists in controlling either the applied voltage or current so that the delivered power is constant throughout the whole ablation process. The last strategy consists in adjusting the delivered power to maintain a certain temperature in one or more catheter spots, where temperature sensors are placed. The last two strategies, constant power and constant temperature, are the most common strategies used in cardiac ablation^4,6–10^ because they are non-dependent on the tissue impedance changes during the ablation process^11^.

When RFA is applied to cardiac tissue, certain potential complications must be considered. A potential complication is the formation of thrombi due to blood coagulation at temperatures above 70 ºC^4^ what can increase the risk of stroke. Other potential complications to be considered are related with temperatures above 100 ºC. At these temperatures, water in blood or in tissue experiments a phase change from liquid to gas. In this situation, so-called steam pops (intramyocardial gas formation) can occur leading to mechanical damage to tissue. Also, gaseous emboli can produce distal problems, specially at the brain. Other potential complications, some of them fatal for patients, which result from undesired thermal damage to adjacent structures and which have been reported during or after radiofrequency ablation procedures, are atrioesophagial fistula, internal bleeding or damage to adjacent nerves^12^.

To minimize the risk of the above mentioned complications when using RFA, in recent years a novel cardiac ablation technique has been developed for the treatment of cardiac arrhythmias: Pulsed Field Ablation^13^ (PFA). PFA is an ablation technique that consists in subjecting the tissue to brief high electric field exposures. Its mechanism of action is thought to be predominantly based on irreversible electroporation (IRE) with very low risk of thermal damage. (In truth, most authors consider that the terms IRE and PFA are synonyms.)

Electroporation is a biophysical phenomenon in which the cell membrane permeability increases when the cell is exposed to electric fields of high intensity and short duration^14^. This increase in permeability occurs if the electric field magnitude exceeds a certain threshold. If, after field exposure, the cell membrane restores its integrity and the cell survives, the phenomenon is called reversible electroporation^15^. On the other hand, when the electric field exceeds a higher threshold, electroporation causes cell death and the phenomenon is termed irreversible electroporation^16^ (IRE). The use of IRE not accompanied by thermal damage to ablate soft tissues was first proposed in 2005^17^. This ablation method is called non-thermal irreversible electroporation (NTIRE) since the increase in temperature it produces as a result of Joule heating is very low and is not the mechanism by which cells die. In fact, these thermal effects, intended in RFA, are tried to be minimized in PFA to avoid possible complications. In this regard, it must me noted that PFA has shown promising results in terms of reducing the risk of injury to collateral tissues such as the phrenic nerve and most critically, the esophagus, or avoiding other commonly observed effects in RFA such as pulmonary veins stenosis^18–23^. Also a recent clinical study has demonstrated that for short (1 or 2 µs) biphasic high-frequency pulses with short interphase and interpulse delays reduces muscle contractions in healthy patients, and also the pain sensation^24^.

There is not much data on the electric field exposure protocols used in clinical PFA treatments. Traditional IRE protocols used for treating solid tumors typically use monophasic square pulses with a duration of 100 µs repeated at 1 Hz^25^. These protocols evoke nerve stimulation resulting in muscle contractions and pain, thus requiring the use of sedation, local or general anesthesia with muscle relaxants^26^. Arena et al.^27^ proposed a new family of IRE protocols in the field of cancer treatment based on the use of high frequency biphasic square pulses (H-FIRE). These IRE protocols are able to reduce muscle contractions while preserving tissue ablation efficacy^28^. In most of the published PFA pre-clinical and clinical studies, protocols consisting of biphasic pulses are mentioned. Regrettably, in the vast majority of cases, these studies do not disclose details on the used waveforms or exposure durations^29^. The lack of published data in this regard makes it necessary to make assumptions in terms of the applied waveforms or the irreversible electroporation electric field thresholds for cardiac tissue. Clinical trials are currently under development comparing RFA and PFA in terms of safety and effectiveness^30^.

The objective of this study is to offer a comparison of the morphology of the lesion produced by both ablation techniques based on computational models. The study evaluates the effect of certain parameters that are specific for the case of the cardiac chamber, such as the blood velocity and its cooling effect, or the catheter orientation. Specifically, the numerical comparison is made for the case of ventricles. Ablation of ventricles, where the wall is considerably thicker than in atrium, is still a challenge when the goal is causing transmural injury. To the best of our knowledge, no previous studies have compared both techniques numerically and in terms of lesion morphology. The study compares the two most common catheter setups used in RFA: monopolar and bipolar catheter configurations.

## Results and discussion

### Reference models comparison

**Figure 1:**
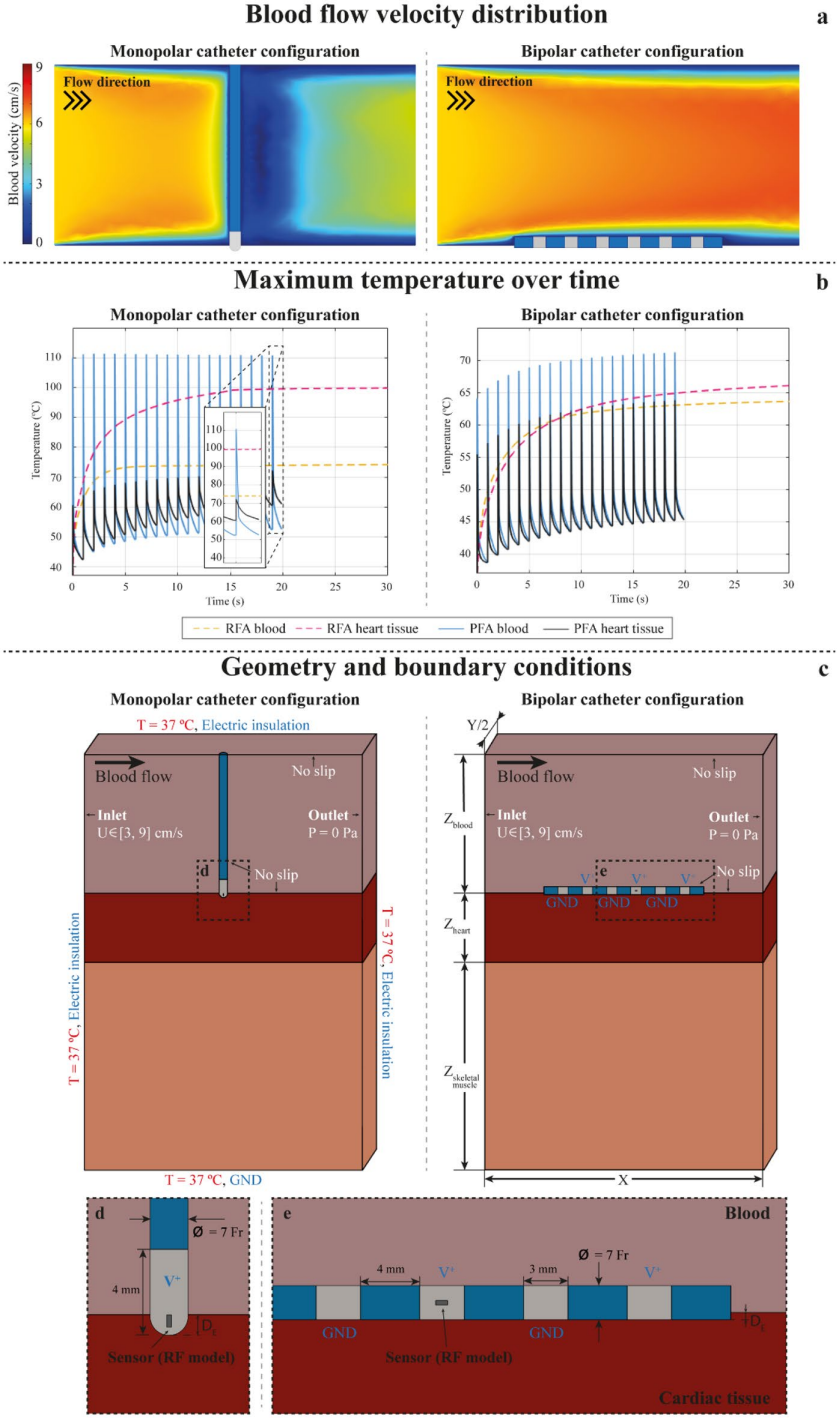
(**a**) Blood flow velocity distributions in (**a.left**) the monopolar catheter configuration and (**a.right**) the bipolar catheter configuration, (**b**) maximum temperature over time in (**b.left**) the monopolar catheter configuration and in (**b.right**) the bipolar catheter configuration. Maximum temperatures predicted for blood and cardiac tissue in RFA (dashed lines) and in PFA (solid lines) and (**c**) geometry and boundary conditions used for the monopolar and bipolar configurations. Electrical (blue), thermal (red) and fluid dynamics (white) boundary conditions are indicated for both catheter configurations. In (**d**) and (**e**) a magnification of the contact points is depicted for the monopolar and bipolar catheter configurations, respectively.

In this section, the results for RFA and PFA models for the reference geometries (see Fig. 1c) and a blood flow velocity of 6 cm/s are shown.

First, the fluid velocity distributions in both catheter configurations are shown at the symmetry plane in Fig. 1a. It can be observed that blood velocity distribution is considerably different for both catheter configurations. In the monopolar configuration, the blood flow has speeds greater than 6 cm/s prior to the catheter and considerably lower speeds after it. This velocity distribution will limit the cooling effect of the blood in motion after the catheter producing an asymmetric effect. In the bipolar configuration, the horizontal disposition of the catheter favors fluid velocities greater than 6 cm/s within the cavity with a much homogenous distribution, however the portion of the surface of the tissue in contact with the fluid is smaller than in the monopolar catheter configuration and this will have an impact on the cooling effect of the blood in the tissue under the catheter.

**Figure 2:**
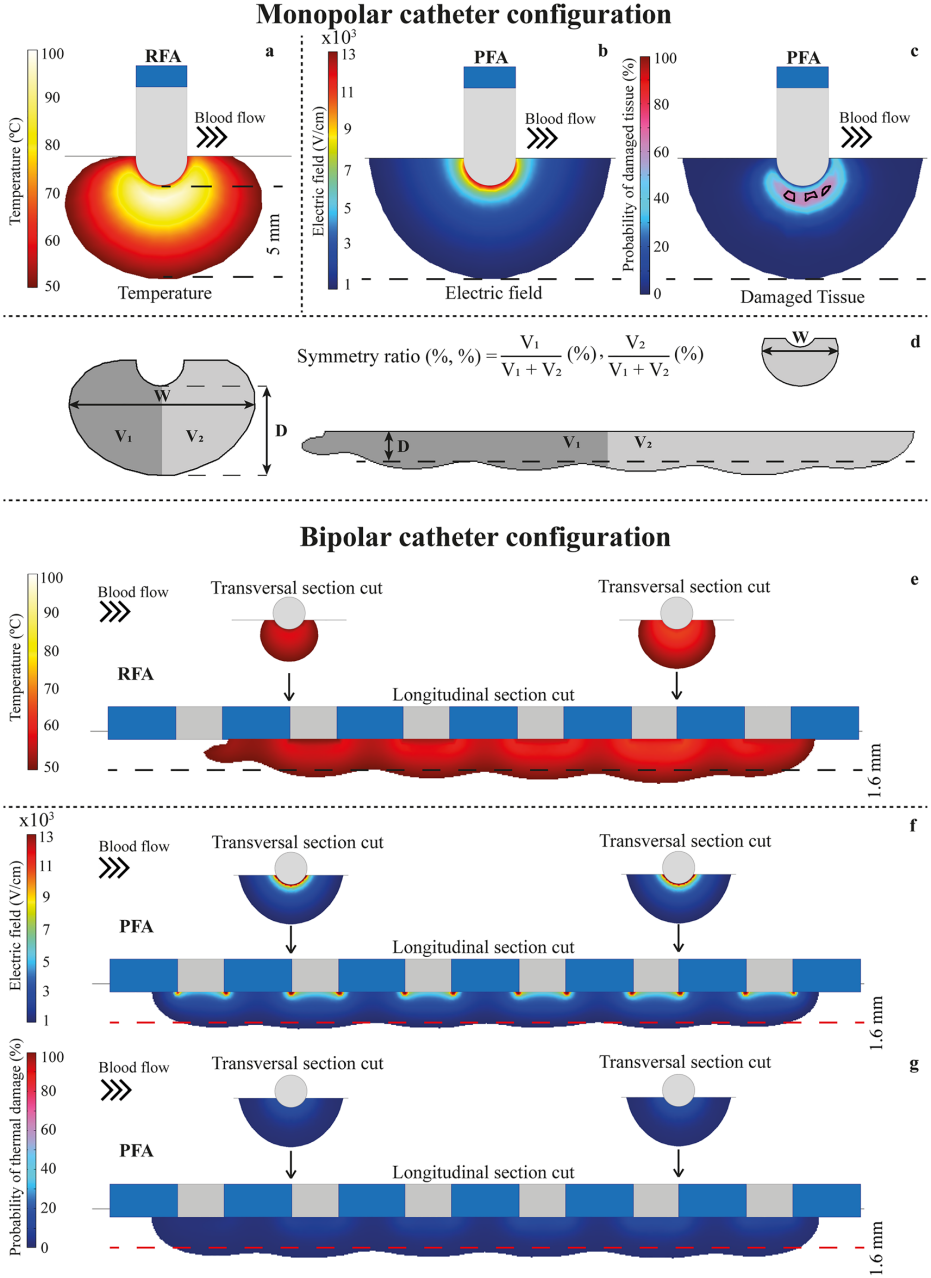
Lesions predicted in the reference models. (**a**)–(**c**): simulations results for the monopolar catheter configuration; (**a**) RFA temperature distribution (displayed data starts at the 50 °C threshold for thermal ablation); (**b**) PFA electric field distribution (displayed data starts at the 1000 V/cm threshold for IRE) and (**c**) probability of thermal damage for PFA. Areas enclosed represents the areas where thermal damage is greater than 63%. In (**d**) the geometrical parameters measured for the lesions in the monopolar catheter configuration are shown. (**e**)–(**g**) Simulation results for the bipolar catheter configuration. (**e**) RFA temperature distribution (displayed data starts at the 50 °C threshold); (**f**) PFA electric field distribution (displayed data starts at the 1000 V/cm threshold) and (**g**) probability of thermal damage for PFA.

In Fig. 2 a summary of the results for the lesions predicted for both ablation techniques are displayed. For the monopolar catheter configuration (see Fig. 2a and 2b, the lesions reach their maximum depth just below the center of the electrode tip. As explained in Materials and Methods (see section Lesion criteria), in order to compare both techniques, the electroporation voltage was adjusted to obtain identical lesion depth than in radiofrequency thermal lesion (5 mm depth obtained for the monopolar configuration and 1.6 mm for the bipolar configuration). The criteria for determining the lesion boundaries were a temperature greater than 50 ºC for RFA and an electric field intensity greater than 1000 V/cm for PFA. The RFA model yielded a narrower lesion than that by the PFA model. In terms of maximum lesion width, RFA model provided a considerably lower width of 9.9 mm compared to 12.1 mm obtained in PFA. Accordingly, the total lesion volume was greater in PFA than in RFA. (See Table 1 for a summary of the lesion measurements.) Regarding lesion shape and symmetry, there are clear differences between both techniques. For RFA, the lesion morphology is quite asymmetric while for PFA the lesion is almost perfectly symmetric. This asymmetry in RFA is produced because the cooling effect of blood in motion is very different at both sides of the catheter. RFA injury is produced by a thermal effect and thus, it will be highly impacted by the cooling effect of blood. On the other hand, electroporation-based tissue damage only depends on the electric field intensity which will not be significantly modified by the cooling effect of blood. Therefore, it is worth noting that although the electrical and thermal properties of materials depend on temperature in both models, in the PFA model the cooling effect of blood was negligible compared to RFA.

**Table 1:** Total volume (V), depth (D), width (W) and symmetry ratio (SR) of the lesions for the reference models displayed in Fig. 2. The width (W) in the bipolar catheter configuration was assessed as the width of the lesion in the transversal section cut.

Lesion parameter	Description	Monopolar catheter configuration		Bipolar catheter configuration	
		RFA	PFA	RFA	PFA
V	Total volume (mm^3^)	320.58	459.54	291.56	435.96
D	Depth (mm)	5	5	1.6	1.6
W	Width (mm)	9.9	12.1	5.1	6.6
SR	Symmetry ratio (%, %)	47.9, 52.1	49.9, 50.1	37.4, 62.6	50.0, 50.0

Additionally, in Fig. 2c the probability of thermal damage caused by the PFA treatment is also shown. Although electroporation is a non-thermal ablation method, collateral Joule heating can induce areas of unwanted thermal damage if excessive energy is applied to the tissue as Faroja et al.^31^ previously reported. In our simulations, the PFA model predicted only a very small area (area enclosed by solid lines in Fig. 2c) where the probability of thermal damage could exceed the 63%, which is usually established as the critical value for thermal damage^32^ (tissue injury integral $\Omega =1$). This result demonstrates that the risk for thermal damage and the associated complications in PFA treatments is very low but not zero.

In Fig. 2e–g, the same data are shown for the bipolar catheter configuration. Observe that for this configuration transversal section cuts at two different catheter planes are also added. In this configuration, the lesion depth obtained along the catheter symmetry plane for the RFA model (temperature of the sensor at 55 ºC) is 1.6 mm. For comparison, and similar to the previous case, the voltage for the PFA model was adjusted to obtain this same depth. In both cases, the models predict continuous lesions along the longitudinal axis of the catheter with different shapes. Again, the lesion volume in PFA is greater than in RFA and the lesion lesions are wider for PFA (see Table 1). With this catheter orientation with respect to blood flow, the RFA model predicts a highly asymmetric lesion due to the influence of blood motion (symmetry ratio = 37.4%, 62.6%) with reduced size in the extreme of the catheter facing the inlet of blood and higher size in the opposite extreme. As in the monopolar configuration, this asymmetry is not present in the PFA lesion.

The previous results correspond to the end of the treatment, i.e., at the end of 30 s in RFA and at the end of 20 s in the case of PFA (20 bursts applied at 1 s of repetition period). In Fig. 1b, the temporal evolution of the maximum temperature in blood and in myocardial tissue during the whole simulation time are shown, for both catheter configurations. In the RFA case it can be observed that the maximum temperature increases continuously (but not linearly) over time, both in blood and in cardiac tissue (higher temperatures in cardiac tissue), for both catheter configurations. The temperature varies from 37 ºC to 74 ºC in blood and from 37 ºC to 101 ºC in cardiac tissue for the monopolar catheter configuration and from 37 ºC to 64 ºC in blood and from 37 ºC to 66 ºC in cardiac tissue for the bipolar catheter configuration. This means that with the monopolar setup the probability of generating blood thrombi (temperatures above 70 ºC) and vapor bubbles (generated when the injured tissue reaches temperatures around 100 ºC) is important.

In PFA, the temperature sharply increases only during the duration of each electric field application (100 µs). As it can be observed Fig. 1b, the maximum temperature that is reached either in blood or in cardiac tissue could be momentarily high. For example, at the end of the simulation (i.e., at the end of the treatment), in the monopolar catheter configuration, the blood reaches a maximum temperature above 100 ºC during the pulse and drops to 53 ºC at the end of the pulse. The cardiac tissue reaches a maximum temperature of 72 ºC during the pulse and drops to 62 ºC at the end. (It must be noted that the displayed values are the absolute maximum values in the geometry and thus, this information should not be used to extract conclusions for the rest of the geometry.) In the case of PFA, the points of maximum heating correspond to the very near vicinity of the electrodes where the electric field is maximum, and the temperature profile rapidly decreased with the distance from the electrode. This implies that the risk of thermal damage, if any, would be restricted to a very small region near the electrodes. This observation suggests that irrigated catheters to avoid any excessive heating near the electrodes could be useful for PFA. Also, these results indicate that, for the particular case of the cardiac cavity, there could be a risk of bubble formation due to evaporation and thrombi formation at the catheter tip and attention in this regard should be paid to the electric field delivery protocols in use.

### Effect of blood velocity

**Figure 3:**
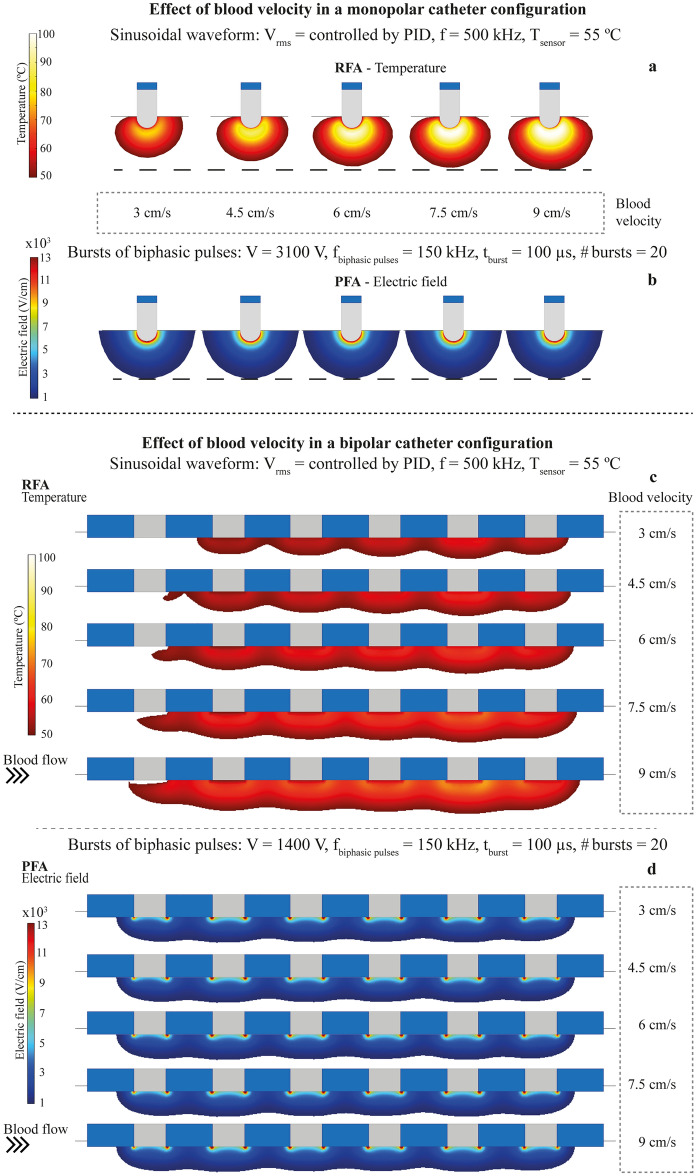
Lesion dependence with blood flow velocity in the monopolar catheter configuration: (**a**) simulated lesion in RFA (temperature greater than 50 ºC) and (**b**) simulated lesion in PFA (electric field magnitude greater than 1000 V/cm); and in the bipolar catheter configuration: (**c**) simulated lesion in RFA (temperature greater than 50 ºC) and (**d**) simulated lesion in PFA (electric field magnitude greater than 1000 V/cm).

The results in this section show the effect that blood motion, inside the cardiac cavity, may have on lesion formation for both ablation techniques. The assayed velocities were in the range from 3 cm/s to 9 cm/s, which are plausible values in the cardiac chamber^33^. In this set of simulations, the input voltage for RFA was adjusted by the proportional-integral (PI) controller for each assayed blood velocity, while for PFA, the voltage amplitude of the applications was kept constant and equal to the value of the reference velocity (6 cm/s). For the monopolar catheter configuration, results in Fig. 3a show how the RFA lesion shape and size change (from 199.60 mm^3^ to 411.47 mm^3^) with blood velocity (see Table 2), for velocities between 3 cm/s and 9 cm/s. The lesion depth ranged from 4 mm to 5.5 mm, getting deeper with blood velocity. The lesion width ranged from 8.6 mm to 10.8 mm and it also grew with blood velocity. The fact that the biggest lesions are produced with the highest blood velocity (highest blood cooling effect) is, at first glance, counterintuitive. This is explained because a temperature-controlled strategy was modelled in these RFA simulations. The temperature sensor was set to a control value (55 ºC). When the velocity is low, the cooling effect of blood in motion is also low, and then, the sensor quickly reaches the control temperature and the energy delivered to the tissue is limited. On the other hand, when velocity increases, the blood cooling effect also increases and the temperature of the sensor takes more time to reach the control temperature allowing the system to deliver more energy to the tissue, producing larger lesion volumes. For PFA, the simulation results in Fig. 3b show very little lesion volume dependence (volume from 452.05 mm^3^ to 485.27 mm^3^; Table 2) on the assessed blood velocities (from 3 cm/s to 9 cm/s.) The lesion depth is 5.0 mm in all cases and the lesion width is 12.1 mm for all velocities. The cooling effect that the blood produces does not significantly affect the electric field distribution for any of the studied velocities.

**Table 2:** Effect of blood velocity on lesion parameters for the monopolar and the bipolar catheter configuration. Total volume (V), depth (D), width (W) and symmetry ratio (SR) of the lesion.

Lesion parameter	Description	Model	Blood velocities				
			3 cm/s	4.5 cm/s	6 cm/s	7.5 cm/s	9 cm/s
**Effect of blood velocity in a monopolar catheter configuration**							
V	Total volume (mm^3^)	**RFA**	199.60	268.20	320.58	366.48	411.47
		**PFA**	485.27	467.59	459.54	455.27	452.05
D	Depth (mm)	**RFA**	4.0	4.6	5.0	5.3	5.5
		**PFA**	5.0	5.0	5.0	5.0	5.0
W	Width (mm)	**RFA**	8.6	9.4	9.9	10.4	10.8
		**PFA**	12.1	12.1	12.1	12.1	12.1
SR	Symmetry ratio (%, %)	**RFA**	45.7, 54.3	47.1, 52.9	47.9, 52.1	48.4, 51.6	48.9, 51.1
		**PFA**	49.7, 50.3	49.8, 50.2	49.9, 50.1	49.9, 50.1	50.0, 50.0
**Effect of blood velocity in a bipolar catheter configuration**							
V	Total volume (mm^3^)	**RFA**	163.92	221.48	291.56	372.18	457.02
		**PFA**	470.17	445.01	435.96	422.71	418.13
D	Depth (mm)	**RFA**	0.9	1.3	1.6	2.0	2.3
		**PFA**	1.6	1.6	1.6	1.6	1.6
W	Width (mm)	**RFA**	2.8	3.4	5.1	4.3	4.8
		**PFA**	6.6	6.6	6.6	6.6	6.6
SR	Symmetry ratio (%, %)	**RFA**	31.8, 68.2	34.4, 65.6	37.4, 62.6	39.8, 60.2	41.6, 58.4
		**PFA**	50.0, 50.0	50.0, 50.0	50.0, 50.0	50.0, 50.0	50.0, 50.0

Regarding symmetry of the lesions, it can be observed that RFA produces non-symmetric lesions with a larger asymmetry ratio for lower blood velocities. In PFA, the morphology of the lesions is symmetric for all the assessed velocities.

The above observations indicate that, in the cardiac chamber, where different blood velocities may be reached depending on the ablation application point^34^, there should be less variability in lesion size and shape for PFA than for RFA. This may represent an important advantage of PFA over RFA.

The same analysis was performed for the bipolar catheter configuration (see Fig. 3c,d). In Table 2 the geometrical features of the lesions are summarized and, similarly to the case of the monopolar setup, the simulations show that PFA is not affected by blood velocity. However, the RFA simulations again predict lesion volume, width, depth and symmetry ratio dependence on blood velocity; all parameters growing with it. As in the monopolar case, lesion asymmetry is more visible for low velocities than for higher ones (see Fig. 3a). In this bipolar setup case, the asymmetry ratio is even higher (31.8 %, 68.2 %) for a blood velocity of 3 cm/s (see Fig. 3c). This higher asymmetry with respect to the monopolar setup could be explained by the fact that, because the bipolar catheter occupies a bigger region, the asymmetry in the cooling effect is more pronounced. It must be noted that the results for the bipolar configuration correspond to an ideal model where the blood flow direction is unique in the whole linear electrode array. This certainly differs from the real situation where for a linear electrode catheter blood flow will be more complex and in different directions at different points, leading to even a more asymmetric lesion. In the next section, the effects of the blood flow direction respect to the catheter are shown.

### Effect of catheter orientation

**Figure 4:**
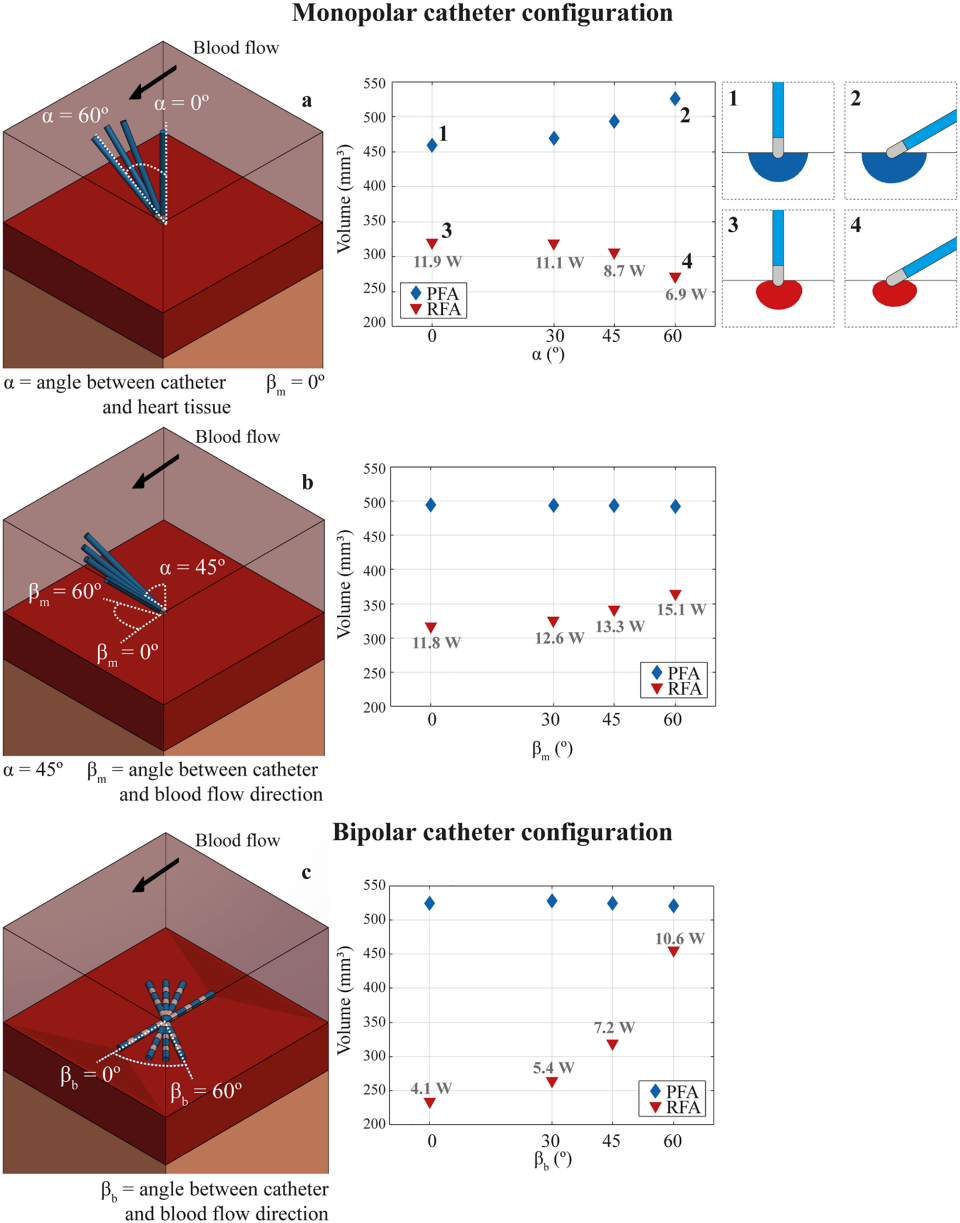
Dependence of the lesion on the angle of the catheter with the cardiac surface (*α*) and with the blood flow (*β*_*m*_ and *β*_*b*_) for (**a**) and (**b**) monopolar and (**c**) bipolar catheter configurations for RFA (inverted red triangle) and PFA (blue diamond). (**a**) Lesion volumes obtained for a fixed value of ${\beta }_{m}=0^\circ$ and varying *α*. Right inset depicts the lesion shapes predicted for (**a.1** and **a.2**) PFA and (**a.3** and **a.4**) RFA for angles $\alpha =0^\circ$ and $\alpha =60^\circ$, respectively. (**b**) Lesion volumes obtained for a fixed value of $\alpha \hspace{0.17em}$= $45^\circ$ and varying ${\beta }_{m}$. (**c**) Lesion volumes obtained for different values of ${\beta }_{b}$(angle between the catheter and the blood flow direction) in the bipolar catheter configuration. The mean power applied (mean value of the complete application time, 30 s) is shown for RFA in all cases.

Another important parameter that can affect the lesion is the orientation of the catheter. In the case of the monopolar catheter configuration, there are two angles to be considered: *α* (angle between the catheter and the cardiac surface, see Fig. 4a) and *β*_*m*_ (angle between the catheter and the direction of blood flow, see Fig. 4b). Simulations at different values of these two angles were performed to study the dependence of the lesions on the catheter relative position to cardiac surface and blood flow direction. The obtained results are summarized in Fig. 4. For the monopolar catheter configuration (Fig. 4a,b), the simulations show that when *β*_*m*_ is fixed to 0º and *α* varies from 0º to 60º, PFA displays a lesion volume growing with *α* (from 459.54 mm^3^ to 525.75 mm^3^; 14.4% increase). In contrast, RFA shows a lesion volume decrease with *α* (from 320.58 mm^3^ to 272.07 mm^3^; 15.13% decrease). In all cases, the lesion volume in RFA is lower than in PFA, being maximum in RFA at the perpendicular position of the catheter ($\alpha =0^\circ$) and in PFA when $\alpha =60^\circ$. These results indicate that both techniques are sensitive to the catheter angle with respect to tissue surface but in an inverse direction. For both techniques, the electric field distribution similarly change with the angle of the catheter with respect to the tissue surface. For PFA, this change directly impacts the shape and size of the lesion. For RFA the impact is more complex because a change in the angle will, on the one hand, modify the resistive heating component due to a change in the electric field distribution and, on the other hand, for the case of a temperature controlled RFA approach, the lesion volume will be also modified because the exposure of the temperature sensor to the blood flow will change, and thus, the power applied to the tissue will depend on this (see mean power applied for each angle in Fig. 4c). Higher exposures of the sensor to blood flow will lead to higher lesion volumes (higher cooling of the sensor by the blood flow resulting in larger energy delivery). The fact that RFA reaches the maximum lesion volume when $\alpha =0^\circ$, and that any deviation from the vertical position is translated in a reduced lesion volume, may be seen as an additional disadvantage of RFA with respect to PFA. This is especially relevant inside the beating cardiac chamber, where maintaining a perfect perpendicular position of the catheter with the tissue surface is highly challenging. Finally, in terms of maximum lesion depth predicted by the model, a reduction of 12% depth was predicted for the worst case ($\alpha =60^\circ$) in RFA, however, no significant depth reduction was predicted in PFA lesions.

For a fixed value of $\alpha =45^\circ$ (see Fig. 4b), the dependence of lesion volume on the angle of catheter with the direction of the blood flow (*β*_*m*_ ranging from 0º to 60º) was assessed. In this case, the simulations predict that the effect of catheter orientation with respect to the blood flow was negligible in PFA, with lesion volumes ranging from 494.67 mm^3^ to 492.25 mm^3^. This is because the direction of blood flow will not significantly impact the overall electric field distribution in the tissue. However, RFA simulations show a high dependence of lesion volume on the blood flow direction that increases with *β*_*m*_: from 317.55 mm^3^ (${\beta }_{m}=0^\circ$) to 365.35 mm^3^ (${\beta }_{m}=60^\circ$). This strong dependence for RFA is again produced by the cooling effect of the blood flow. For ${\beta }_{m}=0^\circ$ the surface of the electrode exposed is minimum (low sensor cooling) and the lowest lesion volume is obtained in this case. When *β*_*m*_ increases, more electrode surface is exposed to blood in motion (high sensor cooling), hence obtaining larger lesion volumes.

Of course, when the information of various temperature sensors is combined, the impact of catheter orientation could be minimized or avoided in sophisticated RFA catheters. However, it is clear that the non-dependence on catheter orientation of PFA represents an additional advantage of PFA over RFA; either because minimizes lesion variability or because reduces the complexity of catheters and application algorithms used.

For the bipolar catheter configuration (see Fig. 4c) where *β*_*b*_ is the angle between the catheter longitudinal axis and the direction of blood flow ranging from 0º (catheter axis parallel to the blood flow direction) to 60º, the results predict a strong dependence of lesion volume with *β*_*b*_ in RFA. The volumes ranged from 235.2 mm^3^ to 455.8 mm^3^, growing with *β*_*b*_. However, the results in PFA show that lesion volume remains invariant regardless of the catheter orientation and the lesion volumes range from 523.9 mm^3^ to 525.1 mm^3^. In this catheter configuration, the difference between the behavior obtained for RFA and PFA is even higher than for the monopolar catheter case. As in the monopolar configuration, the RFA dependence on the orientation may be explained by the variation of the exposure surface of the temperature sensor to the blood flow.

### Study limitations

In this study the effect of the catheter tip contact force on lesion formation was not considered. However, we know that for RFA differences in contact force can cause changes in the contact area between the heart tissue and the catheter tip, thus, modifying the impedance between the tip of the catheter and the tissue and the eventual lesion created^35^. For PFA, recent studies have also shown the impact of contact force for different application approaches^36,37^. In Supplementary Materials, the importance and complexity of contact in lesion formation for both techniques was assessed for a “worst case” contact scenario. Future work should be performed to deeply study this effect. Another limitation of our model is that the simulations only considered one preferential direction of blood flow. This represents an oversimplification of the real situation inside the cardiac chamber where blood has multiple directions^38^; as well as a laminar flow regime was simulated but in the real scenario, turbulent flow may be present in some parts of the cardiac chamber^39^.

As shown, the temperature sensor position is a very relevant parameter when a temperature-controlled strategy is performed in RFA. Most catheter manufacturers do not reveal information regarding the sensor position. The results of the simulations would be certainly impacted if other sensor positions were assayed. Additionally, for simplicity, the presented simulations only included one temperature sensor, but some of the commercially available catheters used nowadays in RFA are more complex and incorporate several temperature sensors. With this increase in complexity, we could speculate that by combining the information of several temperature sensors distributed in the catheter, optimized energy applications may be achieved and the observed effects of catheter orientation may be partially mitigated.

Finally, the use of isotropic parameters in all tissues could lead to an oversimplification of the real problem due to the anisotropic nature of muscle tissues (especially skeletal and cardiac muscle).

## Conclusions

By means of computational models, this study compared radiofrequency ablation (RFA) with pulsed field ablation (PFA) in endocardial catheter ablation. Monopolar and bipolar catheter configurations were assayed under the assumption of blood flow. The simulations results indicate that substantial differences in terms of lesion morphology between RFA and PFA are to be expected. Both for monopolar and bipolar configurations, for the same lesion depth, the simulations predict that PFA lesions are wider and occupy a bigger volume than those obtained in RFA. Also, RFA lesions display a much more asymmetrical morphology compared to that obtained in PFA for all studied cases. Simulated PFA treatments indicate that the risk of collateral thermal damage is very low but not zero, suggesting that this issue should be taken into consideration when designing PFA catheters and pulsing protocols.

The simulation results also indicate that temperature-controlled RFA is highly impacted by blood flow velocity. Shallower, narrower and more asymmetric lesions are obtained for low flow velocities. On the other hand, in PFA blood flow velocity has no substantial impact on the lesion size and morphology.

Finally, the simulation results also indicate important differences regarding the impact of the catheter orientation on the lesions. While for monopolar RFA a perpendicular orientation of the catheter with respect to the heart surface generates larger lesions, in the case of PFA the opposite is obtained; the largest lesions are obtained with the most obliquus catheter position. Furthermore, RFA lesions are impacted by the orientation of the catheter with respect to the blood flow whereas such impact is negligible in the case of PFA lesions.

This study numerically reveals for the first time the differences in terms of lesion morphology between RFA and PFA in the cardiac chamber. Additionally, this study exposes an important advantage of PFA over RFA: its negligible dependence on variations in blood flow velocity and on variations in the orientation of the ablation catheter.

## Methods

### Model geometry

Reciprocal models for radiofrequency ablation (RFA) and pulsed field ablation (PFA) were developed and assayed in this study. The RFA models and the PFA models used the same geometries. The two treatment modalities were evaluated both in monopolar and bipolar catheter configurations assessing the effect of blood in motion and catheter orientations on the lesion morphology. In essence, the geometry of the treated region consisted in a simplified model of the heart cavity which included of a three-layer slab where blood in motion was modelled at the upper layer. Some previous computational studies of RFA with a monopolar catheter configuration, and a similar geometry, only included the blood and the heart tissue with the dispersive electrode at the bottom and in direct contact with the heart tissue^5,40–44^. In clinical RFA, the dispersive electrode in a monopolar catheter configuration is placed on the back of the patient. Having the dispersive electrode in direct contact with heart tissue could lead to an oversimplification of the models. For this reason, we included a layer of skeletal muscle to model the presence of other tissues. Therefore, the geometries used in the models include 4 different domains: the catheter, a layer of blood in motion, a layer of heart tissue (myocardium) and a bottom layer of skeletal muscle (see Fig. 1c).

The dimensions of the treated region model both for RFA and PFA (*X*, *Y*, *Z*_*blood*_ and *Z*_*cardiac*_) were taken from González-Suárez et al.^42^ (see Table 3), who performed a convergence test in order to avoid boundary effects in their RFA models. The thickness of the heart tissue in this geometry is closer to values found in the ventricle than in the atria. The thickness of the skeletal muscle layer (*Z*_*skeletal*_) was set to 60 mm. This value was arbitrarily chosen to account for the different tissues between the heart and the dispersive electrode. In order to save computational cost and simulation time, it was decided to use a semi slab (*Y*/2 instead of *Y*) of the models (see Fig. 1c) when the symmetry of the study allowed it (i.e., in the blood flow velocity sub-study described above).

**Table 3:** Geometrical dimensions of the slabs for RFA and PFA models.

Domains	Dimensions (in mm) of RFA and PFA models		
	Width (X)	Depth (Y)	Height (Z)
Blood	80	80	40
Heart (myocardium)			20
Skeletal muscle			60

Non-irrigated catheters were modelled in this study. For the monopolar catheter configuration, the chosen catheter had a diameter of 7 Fr (2.33 mm). The electrode tip was placed perpendicular to the cardiac surface at the center of the slab. The dispersive electrode was placed at the bottom of the slab (see Fig. 1c). For the bipolar configuration, the electrode array (consecutive ring electrodes) was placed horizontally, centered, and longitudinal to the direction of the blood flow (see Fig. 1c). This bipolar electrode geometry mimics catheter configurations where energy is applied between adjacent ring electrodes forming rings or linear arrays that are more commonly used in atria ablation. Both catheters were placed against the heart surface. Due to the pressure exerted by the tip of the monopolar catheter on the cardiac surface, the penetration depth (*D*_*E*_ ) of the tip was greater in the monopolar catheter configuration (${D}_{E}=1.25$ mm, see Fig. 1d)^40^ than in the bipolar configuration (${D}_{E}=0.5$ mm, see Fig. 1e)^45^, where the contact surface is significantly greater, generating lower penetration in the tissue. In the RFA model, a temperature sensor was modelled inside the electrode. For the monopolar catheter configuration, the size and position of the sensor was similar to those used in Alba-Martínez et al.^46^; for the bipolar catheter configuration, it was placed at the central electrode of the array.

### Modelled physics

Both ablation techniques apply electric currents (with different characteristics) to the tissues, and therefore both generate Joule heating whose thermal consequences may strongly depend on the cooling effect of blood in motion. Consequently, three physics were considered in this study: electric, thermal and fluid dynamics. The models included a double-coupled problem between electric and thermal solutions to model tissue heating by Joule effect, and between thermal and fluid dynamics solutions to model the cooling effect of blood flow in motion.

The electric problem was solved using the AC/DC module of COMSOL Multiphysics solving the following equation:

1 $$\nabla \cdot (\sigma \nabla \Phi )=0$$

where $\sigma$ is the electrical conductivity (S/m) and $\Phi$ is the voltage (V). The electric field $\overrightarrow{E}$ was then calculated as $\overrightarrow{E}=-\nabla \Phi$. For the monopolar catheter configuration, the catheter electrode tip was set at a defined potential the dispersive electrode was set to ground (see Fig. 1c). For the bipolar catheter configuration, the electrodes were set alternatively to a defined potential or ground (see Fig. 1c).

As later described in detail (see section Radiofrequency ablation setup for RFA simulations and section Pulsed field ablation setup for PFA simulations), the electrical conductivity ($\sigma$) of living tissues was modelled to be dependent both on the temperature (for modelling the dependence of the conductivity of ionic solutions with temperature; both in RFA and PFA simulations) and on the electric field magnitude (for modelling for the electroporation phenomenon; only in PFA simulations). Therefore, the solution to the electric problem was dependent on the solution to the thermal problem which in turn was dependent on the solution to the electric problem. That is, both problems were coupled.

To obtain the heating produced by the delivered currents, simplifications were performed for computational reasons. For RFA, sinusoidal waveforms with a frequency of 500 kHz were considered. Since displacement currents are much less important than conduction currents for this frequency, the biological medium can be considered as resistive^42^. Considering this approximation, and to save computational cost, the sinusoidal voltage waveform was modelled as a DC voltage with a magnitude equal to that of the RMS voltage:

2 $${\left.{V}_{RMS}\right|}_{RFA}=\frac{{V}_{P}}{\sqrt{2}}$$

where ${V}_{P}$ is the amplitude of the applied sinusoidal voltage.

In the PFA case, bursts of biphasic square pulses were simulated. For the biphasic square bursts, a similar approximation to save computation costs was performed replacing each of them by a unique monophasic square pulse of similar amplitude:

3 $${\left.{V}_{RMS}\right|}_{PFA}={V}_{P}$$

Within the heart tissue, the governing equation for solving the thermal problem was based on the Penne’s Bioheat equation that was solved using the Heat Transfer module in COMSOL Multiphysics:

4 $${\rho }_{i}{c}_{i}\frac{\partial T}{\partial t}=\nabla \cdot \left(k\nabla T\right)+q-{Q}_{p}+{Q}_{m}$$

where ${\rho }_{i}$ is the density (kg/m^3^), ${c}_{i}$ is the heat capacity (J/kg·K), *i* = *l* represents the liquid phase and *i* = *v* the vapor phase parameters of the biological tissue, $T$ is the temperature (K), $t$ is the time (s), $k$ is the coefficient of heat conductivity (W/m·K), $q$ is the heat source by Joule heating caused by RF energy (W/m^3^) which is proportional to the electrical conductivity and to the square of the electric field magnitude (V/m), $q=\sigma |E{|}^{2}$, ${Q}_{p}$ is the heat loss caused by blood perfusion (W/m^3^) and ${Q}_{m}$ is the metabolic heat generation (W/m^3^). ${Q}_{p}$ was not considered since it has been demonstrated computationally and experimentally that the blood flowing away in the coronary arteries of the heart tissue does not have significant influence on temperature distribution during radiofrequency ablation in cardiac tissue^47^. For PFA, the same assumption was considered. Likewise, ${Q}_{m}$ was not considered because it is negligible in comparison to the other terms during RFA and PFA. An initial temperature for the entire model of 37 ºC was considered and all the exterior boundaries were fixed at the same temperature (see Fig. 1c). To include the phase change of the biological tissue due to water vaporization, the enthalpy method is included in the Penne’s Bioheat equation (Eq. (4)). Enthalpy is related to temperature by the following expression^48,49^:

5 $${\rho }_{i}{c}_{i}\frac{\partial T}{\partial t}=\frac{\partial (\rho h)}{\partial t}=\frac{\partial }{\partial t}\left\{\begin{array}{ll}{\rho }_{l}{c}_{l}\left(T-37\,^\circ{\rm C} \right) & 37\,^\circ{\rm C} \le T\le 99\,^\circ{\rm C} \\ {\rho }_{l}{c}_{l}\left(99\,^\circ{\rm C} -37\,^\circ{\rm C} \right)+\frac{T-99\,^\circ{\rm C} }{100\,^\circ{\rm C} -99\,^\circ{\rm C} }L & 99\,^\circ{\rm C} <T\le 100\,^\circ{\rm C} \\ {\rho }_{l}{c}_{l}\left(99\,^\circ{\rm C} -37\,^\circ{\rm C} \right)+L+{\rho }_{v}{c}_{v}(T-100\,^\circ{\rm C} )& T>100\,^\circ{\rm C} \end{array}\right.$$

where $h$ is the enthalpy, ${\rho }_{l}$ and ${c}_{l}$ are the density and specific heat of cardiac tissue in a liquid phase and ${\rho }_{v}$ and ${c}_{v}$ for the vapor phase, $L$ is the latent heat (2.162·10^9^ J/m^3^) corresponding to the product of water vaporization latent heat (2257 kJ/kg) and water density at 100 ºC (958 kg/m^3^).

The blood was defined as a fluid domain and the catheter, the heart muscle and the skeletal muscle were defined as solid domains. The blood flow was modelled as a laminar flow using the Laminar Flow module of COMSOL Multiphysics solving Navier-Stokes’s equation:

6 $$\frac{\partial u}{\partial t}+u\cdot \nabla u=-\frac{\nabla P}{\rho }+\mu {\nabla }^{2}u$$

where $u$ is the velocity vector of the blood, $P$ is the blood pressure, $\rho$ is the blood density, $\mu$ is the kinematic viscosity. An inlet boundary with normal inflow velocity was used in one wall and an outlet boundary of null pressure was placed at the opposite wall of the cavity. The values of the inlet blood flow velocity ranged from 3 cm/s to 9 cm/s in agreement with values measured in the cardiac cavity^33^. The reference velocity to compare RFA and PFA lesions was 6 cm/s. A no slip condition was applied to the other boundaries of the blood domain, including to the blood-heart muscle and blood-catheter interfaces (see Fig. 1c).

The workflow of the simulations was the following: first, a stationary study was solved for the blood flow in the model. The blood flow velocity distribution solution was considered stationary since the blood density was approximated to be not affected by temperature changes. Subsequently, the electric and thermal problem were solved simultaneously in a time-dependent study. The solution from the stationary blood flow distribution study was coupled with the thermal problem.

The computational model was solved using finite element methods and a PARADISO solver in COMSOL Multiphysics 5.3 software (COMSOL, Burlington, MA, USA, version 5.3a). Simulations were run on a 64-bit PC with an Intel i7 processor with 64 GB of RAM. A tetrahedral mesh was used with 46082 and 45994 elements in the monopolar configuration (RFA and PFA simulations respectively) and 212259 and 212522 elements in the bipolar configuration (RFA and PFA simulations respectively).

### Material properties

The electrical and thermal properties of the materials used in this study are listed in Table 4. Electrical properties were different for both techniques according to the frequency of the applied electric field in each case. The sensor was included only in the RFA models.

**Table 4:** Electrical and thermal properties of the materials used for RFA and PFA models.

Materials	RFA				PFA			
	${\sigma }_{0}$ (S/m)	${k}_{0}$ (W/m °C)	$\rho$ (kg/m^3^)	${c}_{p}$ (J/kg °C)	${\sigma }_{0}$ (S/m)	${k}_{0}$ (W/m °C)	$\rho$ (kg/m^3^)	${c}_{p}$ (J/kg °C)
Electrode—Pt-Ir^40^	4.6 · 10^6^	71	21,500	132	4.6 · 10^6^	71	21,500	132
Insulation—Polyurethane^40^	10^–5^	0.026	70	1045	10^–5^	0.026	70	1045
Skeletal muscle^66^	0.446	0.49	1090	3421	0.373	0.49	1090	3421
Cardiac muscle^66^:								
Tissue	0.281	0.56	1081	3686	0.228	0.56	1081	3686
Vapor			370	2155.92			370	2155.92
Blood^66^	0.748	0.52	1050	3617	0.706	0.52	1050	3617
Sensor^40^	10^–5^	0.038	32	835	–	–	–	–
Frequency	500 kHz				150 kHz			

### Radiofrequency ablation setup

In this study we decided to model temperature-controlled RFA (i.e., constant temperature strategy) as done in González-Suárez et al.^40^ since it was shown that it maximizes the lesions dimensions in ventricular tissue while reducing the probability of steam-pops and thrombus formation^50^. This delivery mode requires one or more temperature sensors at the electrode. The energy delivered to the tissue is controlled by maintaining a constant target temperature at the sensor. In our model, to implement this control strategy, the maximum temperature reached at the sensor region (0.3 mm × 0.3 mm × 0.75 mm) was considered to be the temperature measured by the sensor and the controller was set to maintain it at 55 ºC. A proportional and integral (PI) controller was used to control the applied voltage to the tissue and it was implemented using the external Livelink MATLAB interface (MathWorks, 2020)^40^. The values of the proportional and integral constants used in this study were ${K}_{P}=4.6 V/^\circ C$ and ${K}_{I}=5.8 V/^\circ Cs$. These values were obtained from preliminary simulations where the values were adjusted to reach the targeted temperature (55 ºC) at the sensor in less than 5 s. The temperature at the sensor was sensed every 20 ms^4^. In cardiac RFA, the energy is applied continuously in each application site with durations ranging from 30 s^51^ to 60 s^40,44^. In the present study, applications of 30 s were modelled to reduce the computational cost. The electrical (*σ*) and thermal (*k*) conductivity of the heart tissue were modelled as temperature-dependent functions and were mathematically defined as piecewise functions taken from González-Suárez et al.^40^:

7 $$\sigma \left(T\right)=\left\{\begin{array}{ll}{\sigma }_{0} \mathit{exp}\left(\alpha \left(T-37\,^\circ{\rm C} \right)\right) & 37\,^\circ{\rm C} \le T\le 100\,^\circ{\rm C} \\ 1.371-0.274\left(T-37\,^\circ{\rm C} \right)& 100\,^\circ{\rm C} <T\le 105\,^\circ{\rm C} \\ 1.371\cdot {10}^{-4} & T>105\,^\circ{\rm C} \end{array}\right.$$

8 $$k\left(T\right)=\left\{\begin{array}{ll}{k}_{0}+\Delta k\left(T-37\,^\circ{\rm C} \right) & 37\,^\circ{\rm C} \le T\le 100\,^\circ{\rm C} \\ 0.6356 & T>100\,^\circ{\rm C} \end{array}\right.$$

where ${\sigma }_{0}$ is the electric conductivity at 500 kHz (see Table 4) and $\alpha$ is the temperature exponential growth coefficient of + 1.5%/°C up to 100 °C^52^. When the tissue reaches 100 ºC, to model the tissue desiccation process, the electrical conductivity linearly decreases four folds in the temperature interval from 100 ºC to 105 ºC^53^. The constant *k*_0_ is the thermal conductivity (see Table 4), and it grows linearly with temperature ($\Delta k$) 0.12%/°C up to 100 °C, from where $k$ is kept constant^54^.

### Pulsed field ablation setup

In irreversible electroporation, the lesion is produced when the electric field magnitude exceeds a certain threshold that depends on the rest of the application protocol parameters (pulse duration, waveform, number of pulses, delay between consecutive pulses, etc.). The PFA protocols that have been proposed are very varied. For the specific case of PFA in the ventricle, Sugrue et al.^55^ reported the use of monophasic pulses in a canine ventricle with voltages ranging from 500 V to 1500 V (using a modified 8-mm radiofrequency ablation catheter) with a pulse duration of 90 µs and repetition frequency from 0.83 Hz to 1 Hz. The number of applied pulses ranged from 10 to 100 pulses in a bipolar catheter configuration. Similarly, Livia et al.^56^ used the same catheter in an ex-vivo Langendorff model of canine heart applying 10 monophasic pulses with voltages ranging from 750 V to 2500 V, with a pulse duration of 90 µs repeated 1 Hz in a monopolar catheter configuration. However, in most of recent preclinical and clinical studies, the use of short microsecond-range biphasic square pulses has been reported without providing details on the actual waveform parameters used. (The lack of detailed data on the protocols was justified by the authors on the basis of industrial proprietary data.) As stated in the introduction, the idea of using this type of pulses was first proposed by Arena et al.^27^ as a way to produce irreversible electroporation while avoiding or reducing nerve stimulation.

In this study we decided to model a biphasic protocol based on recent published data on cardiac tissue of rats^57^ and similar to another study in a swine model^58^. The protocol consisted of 20 bursts of biphasic pulses (150 kHz internal frequency, this means 15 biphasic pulses with no delay between pulses), with a conventional duration of 100 µs per burst at a repetition frequency of 1 Hz between consecutive bursts. The applied voltages in PFA were set to get the same lesion depth than in the RFA model for every catheter configuration. As explained in section Modelled physics, for modelling heating caused by PFA and to save computational cost (and since the biological medium can be considered almost totally resistive at this frequency^42^), the bursts of biphasic pulses were replaced by DC pulses with a duration of 100 µs and the same amplitude ${V}_{P}$ (displacement current can be neglected because they do not contribute to Joule heating).

The electrical conductivity was considered temperature-dependent and also electric field-dependent^59^. The expression for the conductivity dependence with the electric field was the following:

9 $$\sigma (E)={\sigma }_{0}+\Delta \sigma exp(-\mathit{exp}\left(-b(\left|E\right|-{E}_{th})\right))$$

where ${\sigma }_{0}$ is the basal electrical conductivity at 150 kHz (see Table 4), $\Delta \sigma$ is the conductivity increment with the electric field (0.1 S/m), $b$ represents the span of the transition zone (0.01 cm/V) and ${E}_{th}$ is the electroporation threshold (500 V/cm) where the conductivity begins to rise. Since there is no data available relative to the electroporation threshold of the heart in vivo, we had to make assumptions. Most of the recent studies dealing with IRE cardiac ablations refer to threshold values extracted from in vitro experiments^60^ that may substantially differ from the actual values required in vivo^61^. In the present simulations we arbitrarily assumed a value of ${E}_{th}$= 500 V/cm because according to the behavior observed in previous studies in other tissues (liver tissue or tumor cells)^62,63^, the value for ${E}_{th}$ at 150 kHz should be higher than the value for DC pulses (usually assumed to be around 400 V/cm).

To model the dependence of the conductivity with the temperature, we followed the same pattern than in the radiofrequency setup (see section Radiofrequency Ablation Setup, Eqs. (7) and (8)):

10 $$\sigma (E,T)=\left\{\begin{array}{ll}\sigma \left(E\right)\left[\mathit{exp}\left(\alpha \left(T-37\,^\circ{\rm C} \right)\right)\right] & 37\,^\circ{\rm C} <T<100^\circ{\rm C} \\ \sigma \left(E\right)\left[2.5728-0.5145\left(T-37\,^\circ{\rm C} \right)\right]& 100\,^\circ{\rm C} <T<105\,^\circ{\rm C} \\ \sigma \left(E\right)\left[2.5728\cdot {10}^{-4}\right] & T>105\,^\circ{\rm C} \end{array}\right.$$

11 $$k\left(T\right)=\left\{\begin{array}{ll}{k}_{0}+\Delta k\left(T-37\,^\circ{\rm C} \right) & 37\,^\circ{\rm C} \le T\le 100\,^\circ{\rm C} \\ 0.6356 & T>100\,^\circ{\rm C} \end{array}\right.$$

### Lesion criteria

In order to compare the lesions created by both techniques, different criteria to establish the conditions where the tissue would be irreversibly damaged were used.

In traditional RFA (application times ranging from 30 s to 60 s), it is commonly considered that tissue is thermally ablated for temperatures greater than 50 ºC. Consequently, the 50 ºC isotherm was used here to delimit the region of tissue ablated by RFA^64^.

In PFA, cell death is a much more complex phenomenon that depends on the electric field magnitude, but also on several different parameters of the exposure such as the total amount of applications (either pulses, cycles or bursts), the waveform and frequency of the applications, the duration of the applications or the interval between consecutive applications. These exposure features define the ‘exposure protocol’, also known as the ‘pulsing protocol’. In numerical models of electroporation, it is usually assumed that, for a given tissue type and a given exposure protocol, all tissue that exceeds a certain electric field intensity threshold (irreversible threshold) is considered damaged. In the present study, due to lack of published data regarding ablation in cardiac tissue for biphasic protocols, the irreversible threshold was arbitrarily established at a level of 1000 V/cm. Considerably lower values are usually considered based on in vitro data for monophasic pulses^60^. In the present case this higher value was considered because, first, in vivo threshold values are higher than those in vitro^61^, and second, the threshold for biphasic waveforms is higher than for monophasic pulses. According to the scarce available data and our own experience, the actual in vivo threshold will likely be lower than that value, hence the present simulations will represent a worst-case scenario. In the PFA models it was not only modelled the (intentional) damage due to IRE but also the (unintentional) thermal damage. Since the pulses in PFA are very intense but have a very short duration, the 50 ºC isotherm criterion would not produce realistic results. To compute thermal damage here it was used the Arrhenius equation^65^, which is a much more accurate estimator of thermal damage for short exposure durations. The percentage of probability of damaged tissue can be predicted using $P(\%)=100\%\cdot (1-exp\{-\Omega \})$, where $\Omega (t)$ was calculated as follows:

12 $$\Omega (t)=\underset{0}{\overset{t}{\int }}A{e}^{-\frac{\Delta E}{RT(\tau )}}d\tau $$

where $A$ is the “pre-exponential factor” (s^*−*1^), $\Delta E$ is the activation barrier (J/mol), $R$ is the gas constant (8.3143 J/mol K) and $T$ is the temperature (K). Tissue injury (due to thermal necrosis) was considered for a probability threshold greater than 63% $(\Omega =1)$^32^. The values of $A$ (2.94·10^39^ s^*−*1^) and $\Delta E$ (2.596·10^5^ J/mol) where taken from Pérez et al.^44^.

For the sake of a fair comparison, the morphologies of the lesions created by RFA and PFA were, compared for equal lesion depth. For that, it was first computed the RFA model (sensor temperature set to 55 ºC and a reference blood velocity of 6 cm/s). Then, the voltage applied in the PFA model was adjusted to obtain the same lesion depth than that obtained by the RFA reference model. The obtained electrode voltages were 3100 V for the monopolar catheter configuration and 1400 V for the bipolar configuration. All these values ensured the same lesion depth in RFA as in PFA. The lesions were compared in terms of total lesion volume (mm^3^), lesion depth (mm), lesion width (mm) and lesion symmetry ratio (%, %). The symmetry ratio was calculated splitting the lesion volume in two domains (see Fig. 2d) and evaluating the percentage of the total volume on each half domain.

## Supplementary Information

Supplementary Information.

## Data Availability

The numerical simulation that support the plots within this paper are not publicly available due to potential industrial protection but are available from the corresponding author on reasonable request.

## Code availability

The codes necessary to reproduce the results in this paper are not publicly available due to potential industrial protection but are available from the corresponding author upon reasonable request.
