# Supplementary material for: A computational comparison of radiofrequency and pulsed field ablation in terms of lesion morphology in the cardiac chamber
Source: Sci Rep. 2022 Sep 27;12:16144. doi: 10.1038/s41598-022-20212-9 (PMC9515184; doi:10.1038/s41598-022-20212-9)
Supplement: Supplementary file 1 — Supplementary Information. [file 41598_2022_20212_MOESM1_ESM.docx]

Supplementary Material

**Additional simulations: no catheter/tissue contact - “worst case” scenario**

In this section we considered a very particular case that may be of interest for the reader. We numerically compared PFA and RFA when the contact between the catheter and the tissue is lost. In these simulations we considered a “worst case” scenario where the catheter was placed 1 mm away from cardiac tissue. The goal of these simulations was to numerically assess how a loss of catheter tissue contact could affect lesion formation with both techniques. Most of recent PFA works claim that this novel ablation modality could be “more forgiving” than RFA in the case of a loss of tissue contact. However, a recent article demonstrated in a preclinical study of ventricular ablation with a monopolar catheter how catheter/tissue contact greatly affected lesion depth^1^. Another recent PFA study demonstrated how it was possible to create lesions even in the absence of direct tissue contact^2^.

The models used in this sub-study are the same than those explained in the main text. The only difference is the geometry used where the catheter tip (or catheter body for bipolar) is placed 1 mm over the cardiac tissue surface.

**Monopolar configuration**

Figure [S1](#Figure_3) shows the volumes of the lesions for both catheter configurations and Table [S1](#Table_1) summarizes the values of lesions dimensions. For a monopolar catheter configuration, the lesions predicted for RFA and PFA are depicted in Figure [S1](#Figure_3).**(a)** and **(b)**, respectively. For PFA, a clear reduction of lesion depth is observed (30 % reduction in lesion depth measured from the tissue/blood interface). However, lesion width is only slightly modified (5 % reduction) and the lesion maintains a very symmetric semispherical shape. Surprisingly, the lesion depth obtained for the RFA simulations is larger than for PFA (11 % reduction in lesion depth measured from the tissue/blood interface). The asymmetry of the RFA lesion is significantly higher in this case due the blood flow velocity that creates a high cooling effect in the catheter/tissue gap area. The volume of the lesion predicted in RFA model (210.91 mm^3^) is slightly lower than predicted in the PFA model (223.92 mm^3^).

If we focus on maximum lesion depth, these results seem to indicate that, in fact, PFA is more vulnerable than RFA to a loss of catheter/tissue contact. This is not a straightforward result and seems to go in the opposite direction of what it could be expected. However, after careful analysis, this result is completely plausible for the specific RFA simulation conditions of this study. The simulation strategy based on a temperature-controlled algorithm implemented in our model is the responsible of this observation. This strategy regulates the energy provided by the electrode by measuring the temperature at the sensor position ^3^. For our model, when a gap is created between the catheter and the tissue, blood flows in this gap creating a cooling effect around the temperature sensor (see Figure [S2](#Figure_1) (left)). This cooling allows more power to be delivered. In fact, when we measured in COMSOL the applied power in this new simulations, we obtained values that were about twice the values obtained in our previous simulations with contact (from 11.9 W to 22.1 W). This increase in power explains why the RFA lesion in the monopolar setup seems to be less affected by the loss of contact. We anticipate that if the numerical comparison was performed with a different position of the sensor (or multiple sensors) or with a constant-power strategy, the observations would be different. This is a limitation of the current study, but comparing all RFA application conditions and strategies is out of the scope of the present study.

**Bipolar configuration**

For the bipolar catheter configuration, lesion shape is shown in Figure [S1](#Figure_3).(**c**) and (**d**). In this case, the lesion depth is lower in RFA (1.2 mm) than in PFA (1.4 mm). Also the volume of RFA is considerably lower (91.49 mm^3^) than in PFA (194.26 mm^3^). A remarkable asymmetry for the RFA lesion is observed in this case.

As observed in Figure [S2](#Figure_1) (right), the blood velocity distribution computed by the model, predicts a negligible effect at the center of the bipolar catheter, which corresponds to the place where the temperature sensor was arbitrarily placed. In this case, the cooling effect of blood around the sensor is negligible and then, the power delivered by the system should be similar to the case of perfect tissue contact (measured values in COMSOL are 4.1 W with contact against 5.3 W without contact). This result demonstrates how for similar applied power values, RFA is more vulnerable than PFA when there is no contact between the catheter and the cardiac tissue.


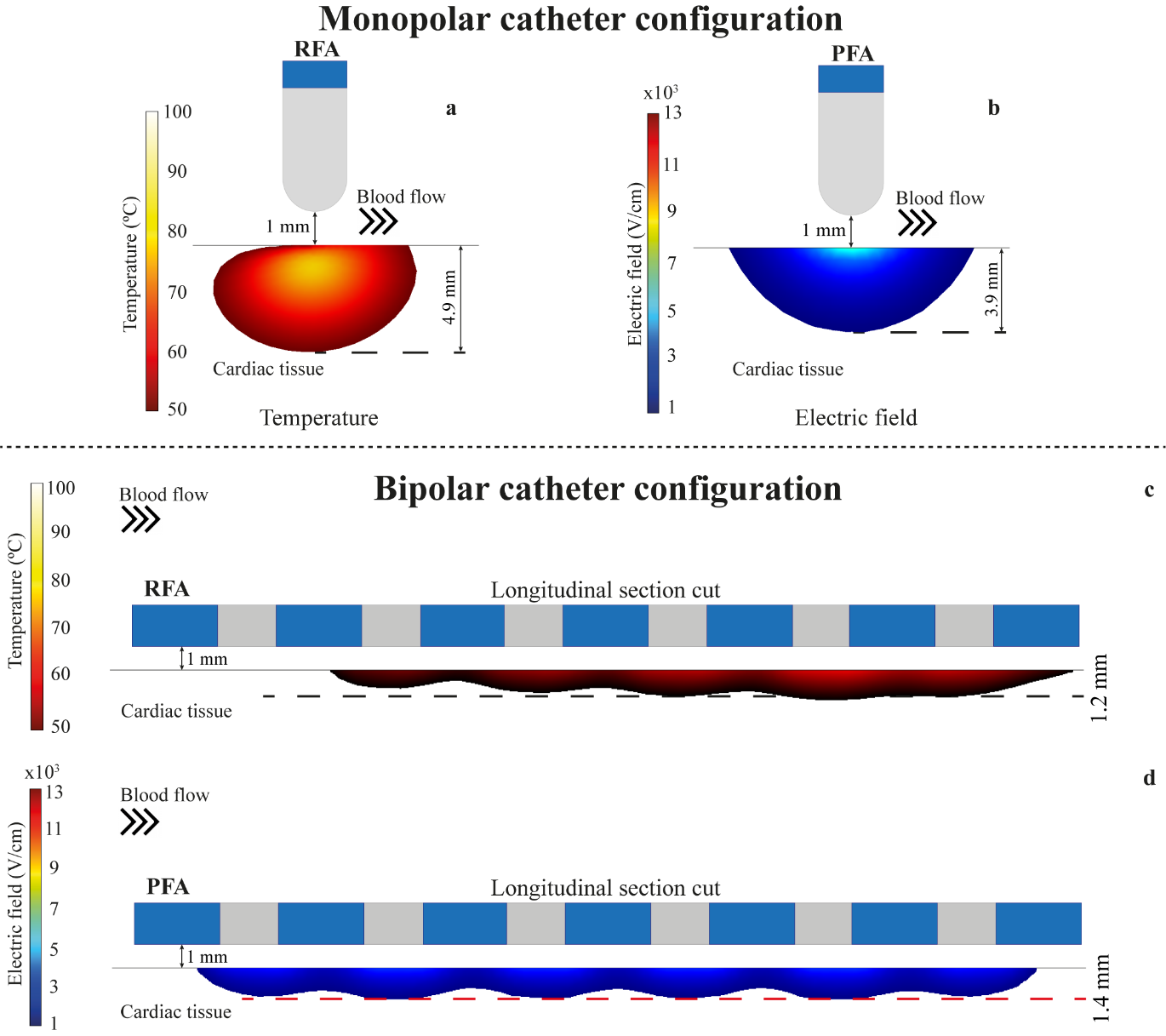


**Figure S1.** Lesions predicted in the reference models for an electrode separation of 1 mm above the cardiac tissue. (**a**) and (**b**): simulations results for the monopolar catheter configuration; (**a**) RFA temperature distribution (displayed data starts at the 50 °C threshold for thermal ablation); (**b**) PFA electric field distribution (displayed data starts at the 1000 V/cm threshold for IRE). In (**c**) and (**d**) simulation results for the bipolar catheter configuration. (**c**) RFA temperature distribution (displayed data starts at the 50 °C threshold); (**d**) PFA electric field distribution (displayed data starts at the 1000 V/cm threshold).

| Lesion  parameter | Description | Monopolar catheter configuration | | Bipolar catheter configuration | |
| --- | --- | --- | --- | --- | --- |
|  |  | **RFA** | **PFA** | **RFA** | **PFA** |
| V | Total volume (mm^3^) | 210.91 | 223.92 | 91.49 | 194.26 |
| D | Depth (mm) | 4.9 | 3.9 | 1.2 | 1.4 |
| W | Width (mm) | 9.4 | 11.4 | 2.7 | 5.7 |
| SR | Symmetry ratio (%, %) | 45.2, 54.8 | 49.9, 50.1 | 22.4, 77.6 | 50.0, 50.0 |

**Table S1.** Total volume (V), depth (D), width (W) and symmetry ratio (SR) of the lesions obtained.


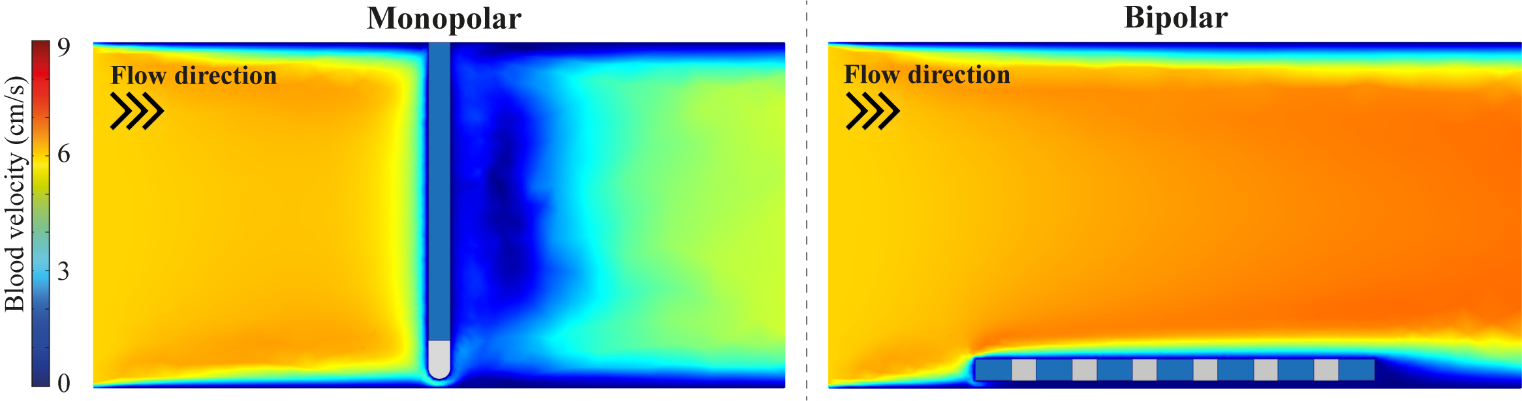


**Figure S2.** Blood flow velocity distributions in (**left**) the monopolar catheter configuration and (**right**) the bipolar catheter configuration for an electrode separation of 1 mm above the cardiac tissue.

# Conclusions

This sub-study numerically demonstrates how contact is a major factor for lesion creation in both ablation modalities. For the case of PFA, a loss of contact is translated in a reduction on lesion depth in the cardiac tissue, but not a complete loss of efficacy. In fact, a recent study showed that the relationship between lesion depth and offset distance was linear^2^. This would suggest that the lesion shape for PFA could be highly predictable even without contact. For the case of RFA, this is much complex and depends on the energy delivery strategy used, the position of the sensors in the catheter (if it is the case), the blood flow velocity, etc. For the specific monopolar setup simulated, the model suggests that a poor contact between catheter and cardiac tissue is less dramatic in terms of lesion depth with RFA using a temperature-controlled strategy For the bipolar setup, PFA is less affected than RFA. These results may not be extended to a general case and deeper and more complex numerical studies specifically aiming at comparing the vulnerability of PFA and RFA for contact should be performed.

# References

1. Nakagawa, H. *et al.* B-PO03-131 EFFECTS OF CONTACT FORCE ON LESION SIZE DURING PULSED FIELD ABLATION. *Hear. Rhythm* **18**, (2021).

2. Verma, A. *et al.* PO-646-02 EFFECTS OF TISSUE PROXIMITY ON CARDIAC LESION FORMATION USING PULSED FIELD ABLATION. *Hear. Rhythm* **19**, S228 (2022).

3. Leshem, E. *et al.* Temperature-Controlled Radiofrequency Ablation Using Irrigated Catheters. *JACC Clin. Electrophysiol.* **6**, 83–93 (2020).
